# Roughage biodegradation by natural co-cultures of rumen fungi and methanogens from Qinghai yaks

**DOI:** 10.1186/s13568-022-01462-2

**Published:** 2022-09-19

**Authors:** Yaqin Wei, Hui Yang, Zhiye Wang, Jiang Zhao, Hongshan Qi, Chuan Wang, Jingrong Zhang, Tao Yang

**Affiliations:** 1 Key Laboratory of Microbial Resources Exploitation and Application of Gansu Province, Institute of Biology, Gansu Academy of Sciences, Lanzhou, 730000 People’s Republic of China; 2 Center for Anaerobic Microbes, Institute of Biology, Gansu Academy of Sciences, No. 197 Dingxi South Road, Lanzhou, 730000 Gansu People’s Republic of China; 3 College of Veterinary Medicine, Gansu Agricultural University, Lanzhou, 730000 People’s Republic of China

**Keywords:** Qinghai yak, Rumen, Fungus, Methanogen, Co-culture, Lignocellulose, Biodegradation

## Abstract

Anaerobic fungus–methanogen co-cultures from rumen liquids and faeces can degrade lignocellulose efficiently. In this study, 31 fungus–methanogen co-cultures were first obtained from the rumen of yaks grazing in Qinghai Province, China, using the Hungate roll-tube technique. The fungi were identified according to morphological characteristics and internal transcribed spacer (ITS) sequences. The methanogens associated with each fungus were identified by polymerase chain reaction-denaturing gradient gel electrophoresis (PCR-DGGE) and 16S rRNA gene sequencing. They were five co-culture types: *Neocallimastix frontalis* + *Methanobrevibacter ruminantium, Neocallimastix frontalis* + *Methanobrevibacter gottschalkii, Orpinomyces joyonii* + *Methanobrevibacter ruminantium, Caecomyces communis* + *Methanobrevibacter ruminantium,* and *Caecomyces communis* + *Methanobrevibacter millerae*. Among the 31 co-cultures, during the 5-day incubation, the *N. frontalis* + *M. gottschalkii* co-culture YakQH5 degraded 59.0%–68.1% of the dry matter (DM) and 49.5%–59.7% of the neutral detergent fiber (NDF) of wheat straw, corn stalk, rice straw, oat straw and sorghum straw to produce CH_4_ (3.0–4.6 mmol/g DM) and acetate (7.3–8.6 mmol/g DM) as end-products. Ferulic acid (FA) released at 4.8 mg/g DM on corn stalk and *p*-coumaric acid (PCA) released at 11.7 mg/g DM on sorghum straw showed the highest values, with the following peak values of enzyme activities: xylanase at 12,910 mU/mL on wheat straw, ferulic acid esterase (FAE) at 10.5 mU/mL on corn stalk, and *p*-coumaric acid esterase (CAE) at 20.5 mU/mL on sorghum straw. The *N. frontalis* + *M. gottschalkii* co-culture YakQH5 from Qinghai yaks represents a new efficient combination for lignocellulose biodegradation, performing better than previously reported fungus–methanogen co-cultures from the digestive tract of ruminants.

## Introduction

Abundant plant biomass is an underused feedstock for bioenergy production. Recently, anaerobic fungi have been reported to efficiently break down lignocellulosic biomass (Young et al. 2018). Studies have shown that anaerobic fungi can produce different extracellular cell–wall-degrading enzymes, including cellulases, hemicellulases, esterases, and multienzyme complexes called cellulosomes, to decompose lignocellulose materials to produce H_2_, formate, acetate, ethanol and CO_2_ (Gilmore et al. 2020; Mi et al. 2009, 2016). The activity of plant cell-wall-degrading enzymes secreted by the anaerobic fungi *Neocallimastix patricciarum* and *Neocallimastix frontalis* is higher than that secreted by commercial *Trichoderma reesei*, *Aspergillus oryzae, Aspergillus nidulans* and *Penicillium pinophilum* used in industry (Dijkerman et al. 1997; Yang and Xie 2010; Cao et al. 2013). Therefore, these fungi have wide application prospects in bioenergy, the feed industry, biogas fermentation and other related fields. Anaerobic fungi are classified in the phylum *Neocallimastigomycota*, which contains one class, *Neocallimastigomycetes*, one order, *Neocallimastigales*, one family, *Neocallimasticaceae* and 18 genera based on flagellum numbers, growth patterns and rhizoid forms (Chang and Park 2020). The whole genome sequences of *Anaeromyces robustus*, *Neocallimastix californiae*, *Pecoramyces ruminantium*, *Piromyces finnis* and *Piromyces* sp. E2 are available in the Joint Genome Institute Database (Chang and Park 2020).

Anaerobic fungi use different carbon sources as substrates for mixed acid fermentation, and the metabolites are mainly formate, acetate, lactate, ethanol, H_2_ and CO_2_ (Li et al. 2016, 2021). Methanogens can use the metabolites of anaerobic fungi, thus eliminating the feedback inhibition effect of the metabolites on growth to accelerate anaerobic fungus reproduction, promote the production of fungal ATP, and improve the activity and yield of enzymes (Wei et al. 2016). Co-cultures of an anaerobic fungus with a methanogen significantly improved their ability to degrade lignocellulosic substrates and produced a large amount of CH_4_ and acetate as fermentation end-products (Jin et al. 2011), which provided a theoretical basis for applying anaerobic fungi in biogas fermentation engineering, along with high activity xylanase and acetate production.

Yak (*Bos grunniens*) is a rare herbivorous ruminant resource in the bovine genus that can adapt to high altitudes, cold, and anoxia **(**Long et al. 2008; Song et al. 2021). Yaks thrive in harsh environments and graze on wild grasses as their main source of nutrition. A large number of unique, complex, and diverse microbial communities in the yak rumen can synergistically degrade low-quality wild herbage and dry, withered cold-season grass to provide yaks with energy and nutrients, making the yak rumen a natural anaerobic fermentation system for efficiently degrading lignocellulose (Huang et al. 2021a, b; Huang et al. 2021a, b). Globally, there are more than 14 million yaks, but China is the centre of origin, with the largest numbers mainly grazing on the Qinghai–Tibetan Plateau at a 3000–6000 m elevation year round. There are approximately 4.9 million heads of yak in Qinghai Province, accounting for the largest share (38%) of the total number of yaks in China (Fan et al. 2021; Sun et al. 2011).

China is known as the yak capital of the world. The number of yaks in Qinghai Province ranks first in China. To date, no reports have been published about fungus–methanogen co-cultures from yaks grazing in Qinghai Province of China. There are a large number of different types of yaks and vegetation in the six main production areas of Qinghai Province. Different kinds of natural herbage eaten by grazing yaks lead to different microbial floras in their rumens of yaks among different areas. Therefore, there are different combinations of natural anaerobic fungus–methanogen co-cultures present in the rumen of grazing yaks in different areas. These fungus–methanogen co-cultures can efficiently degrade lignocelluloses. As there is an urgent need to effectively improve the utilization rate of many types of straws, studies focusing on the yak rumen microbiota are needed. Previous studies have reported some fungus–methanogen co-cultures from the rumen of yaks grazing in Tianzhu Tibetan Autonomous Prefecture in Gansu Province of China, with high fibrolytic enzyme activities (Wei et al. 2015, 2016, 2017). It is necessary to further systematically study fungus–methanogen co-cultures from the rumen of yaks in different areas. These microbial resources have not been fully exploited. We hypothesized that fungus–methanogen co-cultures from the rumen of yaks grazing in Qinghai Province can efficiently degrade lignocelluloses with high fibrolytic enzyme activities. This study unprecedentedly focused on isolating fungus–methanogen co-cultures from the rumen of Qinghai yaks, and further explored their capacity to degrade wheat straw, oat straw, corn stalk, rice straw and sorghum straw.

## Materials and methods

### Animal diet and fungus–methanogen co-cultures isolation

Plateau yak is one of the most valued yak breeds in Qinghai Province, China, and has the genes and characteristics of wild yaks. Xinghai County in Hainan Tibetan Autonomous Prefecture in Qinghai Province of China, at an altitude of 4100 m (34°48′–36°14′ N, 99°01′–100°21′ E), was chosen as the experimental site. In the spring (March), plateau yaks (n = 20, male, 4–5 years) grazing in a pasture in Xinghai County were randomly chosen to isolate single anaerobic fungi. The pasture type was alpine meadow with *Festuca ovina L.* as the main species. The use of animals and the procedure for rumen sample collection were approved by local farms in Hainan Tibetan Autonomous Prefecture of Qinghai Province and the Animal Ethics Committees of the Gansu Academy of Sciences (Gansu, China).

Fresh rumen liquid was collected from the rumen of each yak through a dedicated rumen content collector comprising a stainless steel stomach tube and a tiny vacuum pump. The rumen liquid samples were quickly inoculated into anaerobic tubes containing 9.0 mL basal anaerobic fungus medium and 100 mg air-dried chopped wheat straw, which were autoclaved at 121 °C for 20 min. After inoculation, 1600 IU/mL penicillin and 2000 IU/mL streptomycin were added to kill bacteria, and then the culture was placed at 39 °C. The basal anaerobic fungus medium comprised (per litre): yeast extract, 1.0 g; tryptone, 1.0 g; NaHCO_3_, 7.0 g; resazurin (1.0 g/L), 1 mL; yak rumen fluid without cells centrifuged at 10,000×*g* for 15 min at 4 °C, 170 mL; Salt solution I, 165 mL; Salt solution II, 165 mL; l-cysteine hydrochloride, 1.7 g; and distilled water to 1000 mL. Salt solution I contained NaCl, 6.0 g/L; (NH_4_)_2_SO_4_, 3.0 g/L; KH_2_PO_4_, 3.0 g/L; CaCl_2_·2H_2_O, 0.4 g/L and MgSO_4_·2H_2_O, 0.6 g/L. Salt solution II contained 4.0 g/L K_2_HPO_4_.

After several subcultures, the single anaerobic fungi were isolated using the Hungate roll-tube technology. Bacterial contamination in each culture was checked by PCR with the primers 968f/1401r (Su et al. 2008). The formation of CH_4_ was examined using headspace gas chromatography to ensure that methanogens were present in each co-culture. By this method, we isolated single anaerobic fungi and obtained fungus**–**methanogen co-cultures. The cultures were transferred every 4 days to anaerobic fungus medium with 1% (w/v) wheat straw at 39 °C.

### Identification of fungus–methanogen co-cultures and phylogenetic analysis

The cultures incubated for 2 to 3 days in Hungate agar tubes were observed under a light microscope. The cultures incubated in anaerobic fungus liquid medium with or without straw were observed under a phase contrast microscope (Leica, DMIL-PH1, Germany). The anaerobic fungi were first identified according to their morphological features.

The cultures incubated in 0.1% glucose (w/v) liquid medium without straw at 39 °C for 4 days were collected by centrifugation at 10,000×*g* at 4 °C to extract total genomic DNA. The collected pellets were finely ground using liquid nitrogen. DNA extraction was performed using the Fast DNA SPIN Kit for soil (MP Biomedical, Solon, OH, United States) according to the manufacturer’s instructions. Cell lysis in this kit was performed with sodium phosphate buffer and MT buffer in Lysing Matrix E tubes using a Precellys 24 bead beater for 40 s at a speed of 6.0 m/s.

The ITS sequence of the anaerobic fungi was amplified with the forward primer GM1 (5′-TGTACACACCGCCCGTC-3′) and reverse primer GM2 (5′-CTGCGTTCTTCATCGAT-3′) as reported by Li and Heath. The diversity of methanogens in each co-culture was analysed by the polymerase chain reaction–denaturing gradient gel electrophoresis (PCR–DGGE) method, with the primers 519f/915r GC according to Cheng et al. (2009). The 16S rDNA of methanogens was amplified to identify them using the primers Met86F (5′-GCTCAGTAACACGTGG-3′) and Met1340R (5′-CGGTGTGTGCAAGGAG-3′), and the amplification conditions of the Met primers were modified according to Wei et al. (Wei et al. 2017). All sequences of PCR products were determined by Beijing Genomics Institute (BGI) and all sequences were submitted to the GenBank database.

The amplified products were all visualized and purified by 1% agarose gel electrophoresis. The sequences obtained were aligned by using ClustalX V1.83. A Basic Local Alignment Search Tool (BLAST) search was performed with the obtained sequences to determine the homology with sequences already available in the GenBank database. The evolutionary relationships of the methanogens were plotted using the neighbour-joining method. Phylogenetic analyses and phylogenetic trees construction were conducted in MEGA 7.0.

### Scanning electron microscopy

The fungus cultures grown in anaerobic medium containing 100 mg chopped straw at 1% (w/v) at 39 °C for 72 h were centrifuged at 1000×*g* for 5 min. The precipitate was rinsed three times with phosphate-buffered saline (PBS, pH 7.2). The samples for scanning electron microscopy (SEM) observation were prepared according to Wei et al. (2016).

### Screening of co-cultures

According to a number of previous studies, the lignocellulose degradation capability of the fungus–methanogen co-cultures was positively correlated with gas production (Jin et al. 2011; Wei et al. 2015; Getachew et al. 2004; Gasmi-Boubaker et al. 2005; Sebata et al. 2011). Thus, the fungus–methanogen co-culture with wheat straw as the substrate with the highest gas production during the 5-day culture period was selected as the optimum co-culture, and its capacity for degrading five straws and their respective fermentation end-products were determined.

### Experimental design and sampling

Wheat straw, oat straw, corn stalk, rice straw and sorghum straw were used as substrates, respectively. All substrates were sun-dried, chopped and ground to pass through a 2 mm screen. The inocula were the fungus–methanogen co-culture and its fungus pure culture obtained by adding chloramphenicol (50 μg/mL final concentration) to inhibit methanogens. Medium without the inoculum was used as the control. Each substrate (1 g) was added to 123 anaerobic bottles, each containing 90 mL basal medium. The 10-mL inoculum was added to basal medium sparged with highly purified CO_2_ (Li et al. 2020a, b; Solomona et al. 2016; Henske et al. 2017; Ferraro et al. 2018; Hanafy et al. 2018; Joshi et al. 2018; Peng et al. 2018) and supplemented with 1600 IU/mL penicillin and 2000 IU/mL streptomycin. The cultures were incubated at 39 °C for 5 days. During the 5-day incubation, 3 bottles of the fungus–methanogen co-culture were taken out daily to determine end-products, such as CH_4_ and acetate, as well as xylanase, carboxymethyl cellulase (CMCase), filter paper ase (FPase), ferulic acid esterase (FAE), acetyl esterase (AE), and *p*-coumaric acid esterase (CAE) activities, ferulic acid (FA), *p*-coumaric acid (PCA), vanillic acid (VA) and protocatechuic acid (PA) releases. And 3 bottles of the fungus pure culture were taken out daily to determine end-products. The pellets of the centrifuged cultures were collected for the analysis of in vitro dry matter digestibility (IVDMD) and neutral detergent fibre digestibility (NDFD) by the fungus–methanogen co-culture on the five straws.

### Enzyme profile assays

The cultures were centrifuged at 5000×*g* for 10 min at 4 °C to obtain the supernatants to determine xylanase, CMCase, FPase, FAE, AE and CAE activity according to Wei (2016), Cao (2011) and the state standard of the People's Republic of China (GB/T 23874-2009). One unit of enzyme activity was defined as the amount of enzyme that released 1.0 μmol xylose, glucose, FA, *p*-nitrophenol, or PCA per minute per millilitre at pH6.8 and 39 °C.

### Fibre digestibility determination

According to AOAC (2005) Official Methods of Analysis (18th Association of Analytical Chemists, Washington DC, USA), the samples in each bottle were centrifuged and dried at 105 °C for 24 h to determine IVDMD. NDF contents were determined as described by Van Soest et al. Alpha amylase was not used, but sodium sulfite was added to each sample for the NDF assay. The calculation was as follows: IVDMD or NDFD (%) = [(initial DM or NDF of the feed taken for incubation − DM or NDF of the residue)*/*(initial DM or NDF of the feed taken for incubation)] × 100. The dried residue and NDF content of the dried residue in the uninoculated control were taken as the initial DM and NDF values, respectively.

### Phenolic acid release measurement

The extraction methods of FA, PCA, VA and PA from the supernatants of cultures and high-performance liquid chromatography (HPLC) analyses were performed according to the newly improved HPLC method reported by Wang et al. (2013)_._

### Total gas production and CH_4_ measurement

The cumulative gas production during the 5-day incubation was measured by an AGRS-III automated trace gas recording system for real-time detection of anaerobic microbial growth. CH_4_ was measured by gas chromatography (GC), with an HP-Innowax (19091N-133) capillary column, high purity nitrogen as the carrier gas, and a hydrogen flame ionization detector. The determination conditions of CH_4_ were established as follows: total pressure 130 kPa, total flow rate 30.2 mL/min, column flow rate 1.7 mL/min, linear velocity 39.8 cm/s, column temperature 80 °C, gasification chamber temperature 100 °C, and detection chamber temperature 120 °C.

### End-product analysis

The cultures were centrifuged at 10,000×*g* for 10 min at 4 °C to obtain the supernatants. The formate and acetate concentrations in the supernatants were determined by HPLC (Water 2489, USA) on an instrument equipped with an Agilent SB-Aq chromatography column (26 mm × 250 mm, 5 μm, Agilent, USA), a 2489UA detector, a 7725i manual injector (Rheodyne, USA), and a UV2450 ultraviolet–visible spectrometer (Hitachi, Japan). The determination conditions were as follows: mobile phase 5 mmol/L KH_2_PO_4_-H_3_PO_4_ buffer solution (pH = 2.4), flow velocity 0.5 mL/min, detection wavelength 214 nm, column temperature 25 °C, and injection volume 20 μL. The l-lactate and d-lactate concentrations in the supernatants were analysed using an l,d-lactate Assay Kit (Nanjing Jiancheng Institute of Biological Engineering, Nanjing, China). The ethanol concentration was determined using gas chromatography according to the modified method of Boonchuay et al. (2021).

### Statistical analysis

Data are shown as the mean ± standard deviation (SD) and were analysed using one-way analysis of variance (ANOVA) follwed by Tukey’s test with SPSS 18.0 software (Microsoft). *p* < 0.05 was considered to indicate a statistically significant difference.

## Results

### Isolation and identification of fungus–methanogen co-cultures from Qinghai yaks

The gas chromatography analysis showed that all isolated fungus–methanogen co-cultures produced CH_4_, which confirmed the presence of methanogens in each co-culture. Bacterial specific-PCR amplification showed no detectable bacteria in any co-culture. In this study, 31 fungus–methanogen co-cultures were obtained from the rumen of plateau yaks grazing in Hainan Tibetan Autonomous Prefecture in Qinghai Province, China (Table 1). Among the 31 co-cultures, there were 5 types: *N. frontalis* + *M. ruminantium*, *N. frontalis* + *M. gottschalkii*, *O. joyonii* + *M. ruminantium, C. communis* + *M. ruminantium,* and *C. communis* + *M. millerae.* They were identified and named *N. frontalis* + *M. ruminantium* co-culture YakQH1-YakQH4, *N. frontalis* + *M. gottschalkii* co-culture YakQH5-YakQH16, *O. joyonii* + *M. ruminantium* co-culture YakQH17-YakQH23, *C. communis* + *M. ruminantium* co-culture YakQH24-YakQH26, and *C. communis* + *M*. *millerae* co-culture YakQH27-YakQH31. Each anaerobic fungal strain was symbiotic with a methanogen species. The fungal isolates were named *N. frontalis* YakQH1-YakQH16, *O. joyonii* YakQH17-YakQH23, and *C. communis* YakQH24-YakQH31, and the methanogen isolates were named *M. ruminantium* YakQH1-YakQH4, *M. gottschalkii* YakQH5-YakQH16, *M. ruminantium* YakQH17-YakQH23, *M. ruminantium* YakQH24-YakQH26, and *M*. *millerae* YakQH27-YakQH31 (Table 1).

**Table 1:** Classification of the 31 fungus–methanogen co-cultures isolated from the rumen of Qinghai yaks

Co-cultures	Fungus species	GenBank accession no	Fungus morphological description						
			Thallus	Growth nature	Zoospore flagellum	Mycelia	Rhizoid	Associated methanogen species	GenBank accession no
YakQH1	*N. frontalis*	MH482796	M	En	PF	US	B	*M. ruminantium*	MH443285
YakQH2	*N. frontalis*	MH482797	M	Ex	PF	US	B	*M. ruminantium*	MH443286
YakQH3	*N. frontalis*	MH482798	M	En	PF	US	B	*M. ruminantium*	MH443287
YakQH4	*N. frontalis*	MH482799	M	En	PF	US	B	*M. ruminantium*	MH443288
YakQH5	*N. frontalis*	MH482800	M	En	PF	US	B	*M. gottschalkii*	MH443289
YakQH6	*N. frontalis*	MH482801	M	En	PF	US	B	*M. gottschalkii*	MH443290
YakQH7	*N. frontalis*	MH482802	M	En	PF	US	B	*M. gottschalkii*	MH443291
YakQH8	*N. frontalis*	MH482803	M	En	PF	US	B	*M. gottschalkii*	MH443292
YakQH9	*N. frontalis*	MH482804	M	En	PF	US	B	*M. gottschalkii*	MH443293
YakQH10	*N. frontalis*	MH482805	M	Ex	PF	US	B	*M. gottschalkii*	MH443294
YakQH11	*N. frontalis*	MH482806	M	Ex	PF	US	B	*M. gottschalkii*	MH443295
YakQH12	*N. frontalis*	MH482807	M	En	PF	US	B	*M. gottschalkii*	MH443296
YakQH13	*N. frontalis*	MH482808	M	En	PF	US	B	*M. gottschalkii*	MH443297
YakQH14	*N. frontalis*	MH482809	M	En	PF	US	B	*M. gottschalkii*	MH443298
YakQH15	*N. frontalis*	MH482810	M	En	PF	US	B	*M. gottschalkii*	MH443299
YakQH16	*N. frontalis*	MH482811	M	En	PF	US	B	*M. gottschalkii*	MH443300
YakQH17	*O. joyonii*	MH482812	P	Ex	PF	S	HB	*M. ruminantium*	MH443301
YakQH18	*O. joyonii*	MH482813	P	Ex	PF	S	HB	*M. ruminantium*	MH443302
YakQH19	*O. joyonii*	MH482814	P	Ex	PF	US	HB	*M. ruminantium*	MH443303
YakQH20	*O. joyonii*	MH482815	P	Ex	PF	S	HB	*M. ruminantium*	MH443304
YakQH21	*O. joyonii*	MH482816	P	Ex	PF	S	HB	*M. ruminantium*	MH443305
YakQH22	*O. joyonii*	MH482817	P	Ex	PF	US	HB	*M. ruminantium*	MH443306
YakQH23	*O. joyonii*	MH482818	P	Ex	PF	US	HB	*M. ruminantium*	MH443307
YakQH24	*C. communis*	MH482819	M	Ex	UF	–	G	*M. ruminantium*	MH443308
YakQH25	*C. communis*	MH482820	M	Ex	UF	–	G	*M. ruminantium*	MH443309
YakQH26	*C. communis*	MH482821	M	Ex	UF	–	G	*M. ruminantium*	MH443310
YakQH27	*C. communis*	MH482822	M	Ex	UF	–	G	*M. millerae*	MH443311
YakQH28	*C. communis*	MH482823	M	Ex	UF	–	G	*M. millerae*	MH443312
YakQH29	*C. communis*	MH482824	M	Ex	UF	–	G	*M. millerae*	MH443313
YakQH30	*C. communis*	MH482825	M	Ex	UF	–	G	*M. millerae*	MH443314
YakQH31	*C. communis*	MH482826	M	Ex	UF	–	G	*M. millerae*	MH443315

P, polycentric; M, monocentric; Ex, exogenous; En, endogenous; PF, ployflagellated; UF, uniflagellated; S, segmented; US, unsegmented; HB, highly branched; B, branched; G, globular

**Fig. 1:**
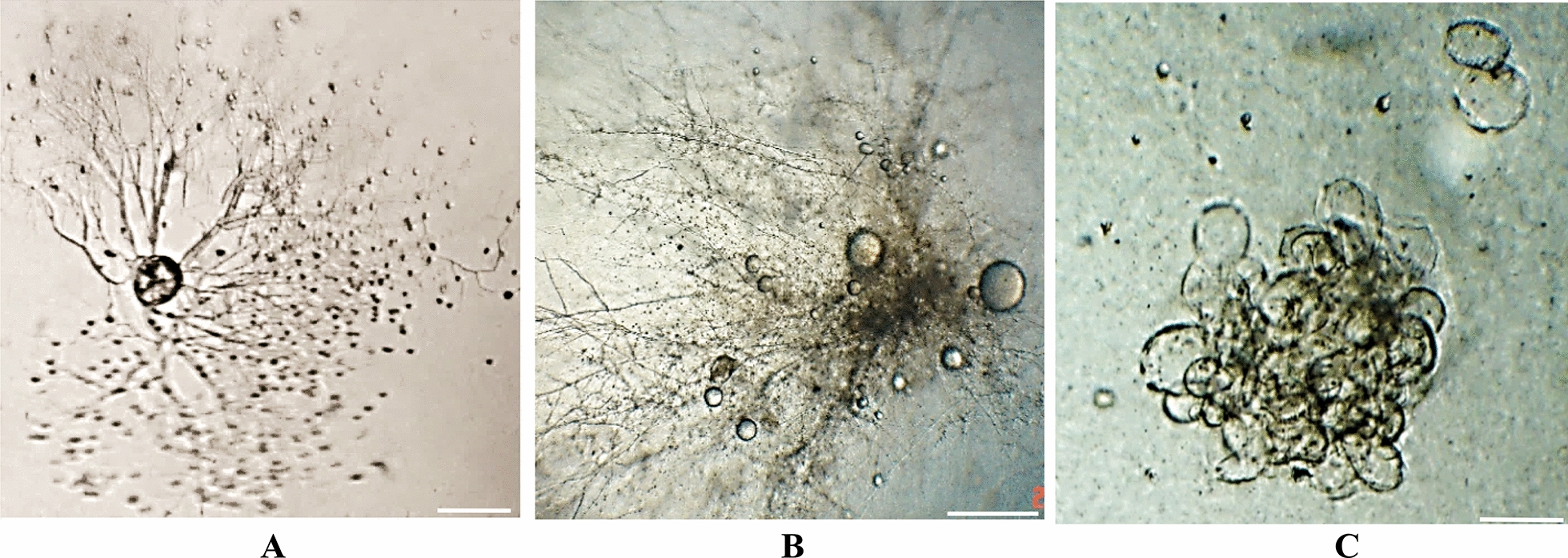
Morphological diversity of anaerobic fungi isolates in Hungate agar roll-tubes. **A** The fungus *N. frontalis* YakQH5 with an endogenous sporangium and a network of rhizoids. **B** The fungus *O. joyonii* YakQH17 produced a rhizomycellium complex with an extensive network of hyphae, single or branched sporangiophores developed from the hyphae, and several globose sporangia full of zoospores were produced from the sporangiophores. **C** The fungus *C. communis* YakQH24 colony. Bars = 100 μm

**Fig. 2:**
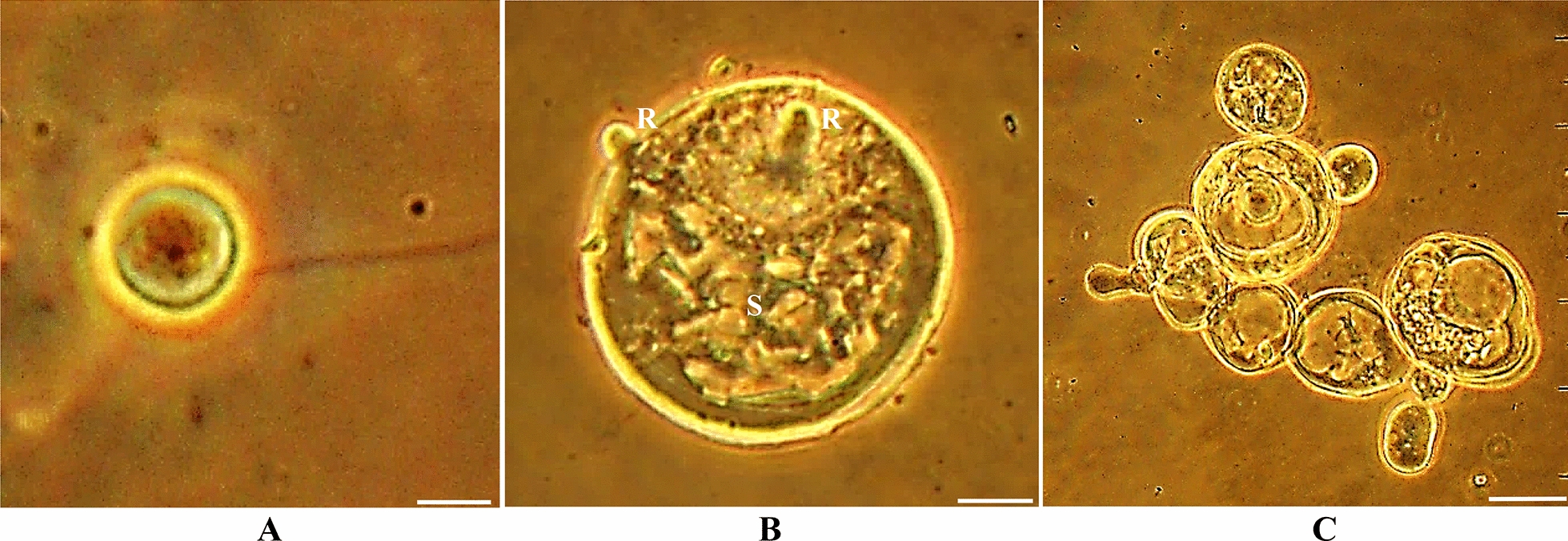
Growth stages of the fungus *C. communis* YakQH24 in liquid glucose culture medium under phase -contrast microscopy. **A** A live uniflagellate zoospore. Bars = 10 μm. **B** The fungus *C. communis* YakQH24 showed bulbous rhizoids after 24 h incubation. Bars = 10 μm. **C** The *C. communis* YakQH24 produced more bulbous rhizoids after 96 h incubation. Bars = 50 μm

**Fig. 3:**
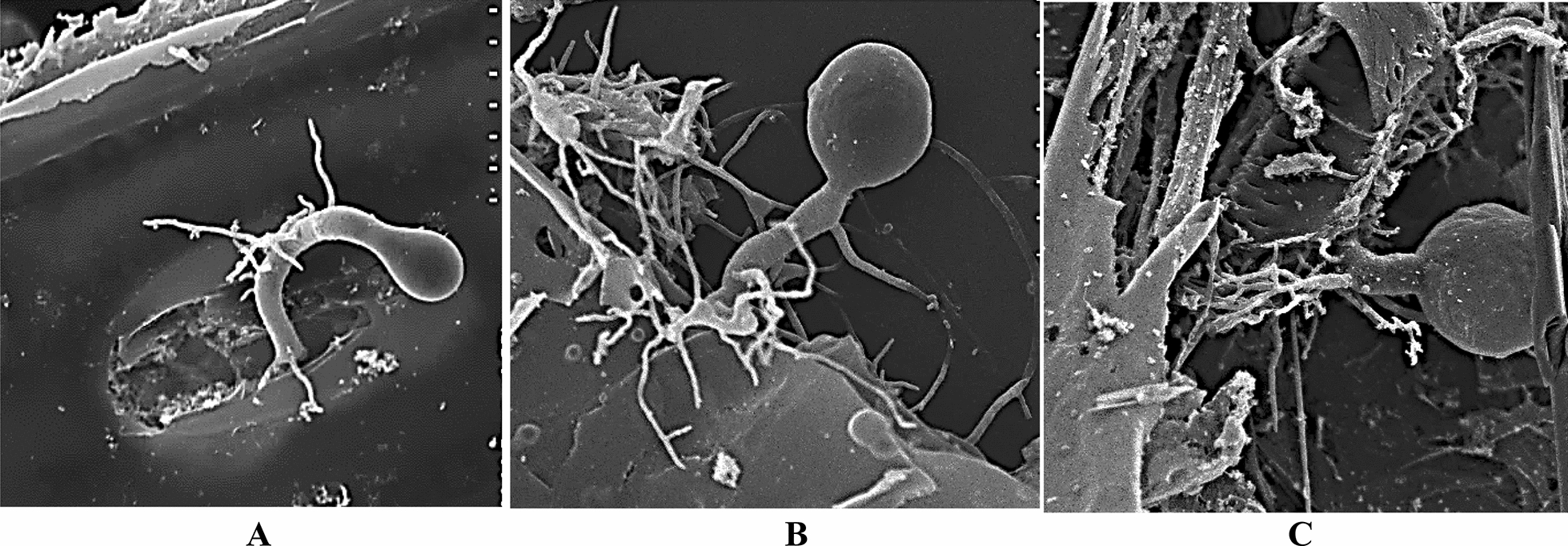
SEM of the anaerobic fungus *N. frontalis* YakQH5. **A** The mycelium of the *N. frontalis* YakQH5 penetrated into the thick-walled tissue of wheat straw after 24 h incubation. **B** The *N. frontalis* YakQH5 grew with an endogenous sporangium growth after 48 h incubation. **C** The dense hyphae of the *N. frontalis* YakQH5 fully penetrated into the wheat straw tissue to physically degrade lignocellulose after 96 h incubation. Bars = 20 μm

The 31 fungi in the co-cultures were observed and identified using light microscopy, phase contrast microscopy, and SEM according to morphological characteristics and the number of zoospore flagella (Figs. 1, 2, 3). The fungal isolates *N. frontalis* YakQH1-YakQH16, *O. joyonii* YakQH17-YakQH23, and *C. communis* YakQH24-YakQH31 showed the morphological features of *N. frontalis, O. joyonii,* and *C. communis,* respectively. The ITS sequences of the 31 fungi in the co-cultures were deposited in GenBank under accession numbers MH482796-MH482826 (Table 1). Through sequence homology comparison with the NCBI database, among the 31 co-cultures, all fungal isolates had a similarity of 99%–100% with *N. frontalis* strain Yak16, *O. joyonii* strain Yak1, and *Caecomyces* sp. AGRL-11 registered in GenBank.

The DGGE results confirmed that one fungus was natively associated with only one methanogen in each co-culture. The 16S rRNA gene sequences of the 31 methanogens in the co-cultures were deposited in GenBank under accession numbers MH443285-MH443315 (Table 1). All methanogen isolates (YakQH1-YakQH31) in the 31 co-cultures belonged to *Methanobrevibacter* sp. based on the 16S rRNA gene sequences. Through sequence homology comparison with the NCBI database, among the 31 co-cultures, all methanogen isolates were found to have a similarity of 99%-100% with *M. ruminantium* strain YakM2, *M. gottschalkii* strain PG, and *M. millerae* strain ZA-10 registered in GenBank.

### Phylogenetic tree construction of methanogens

**Fig. 4:**
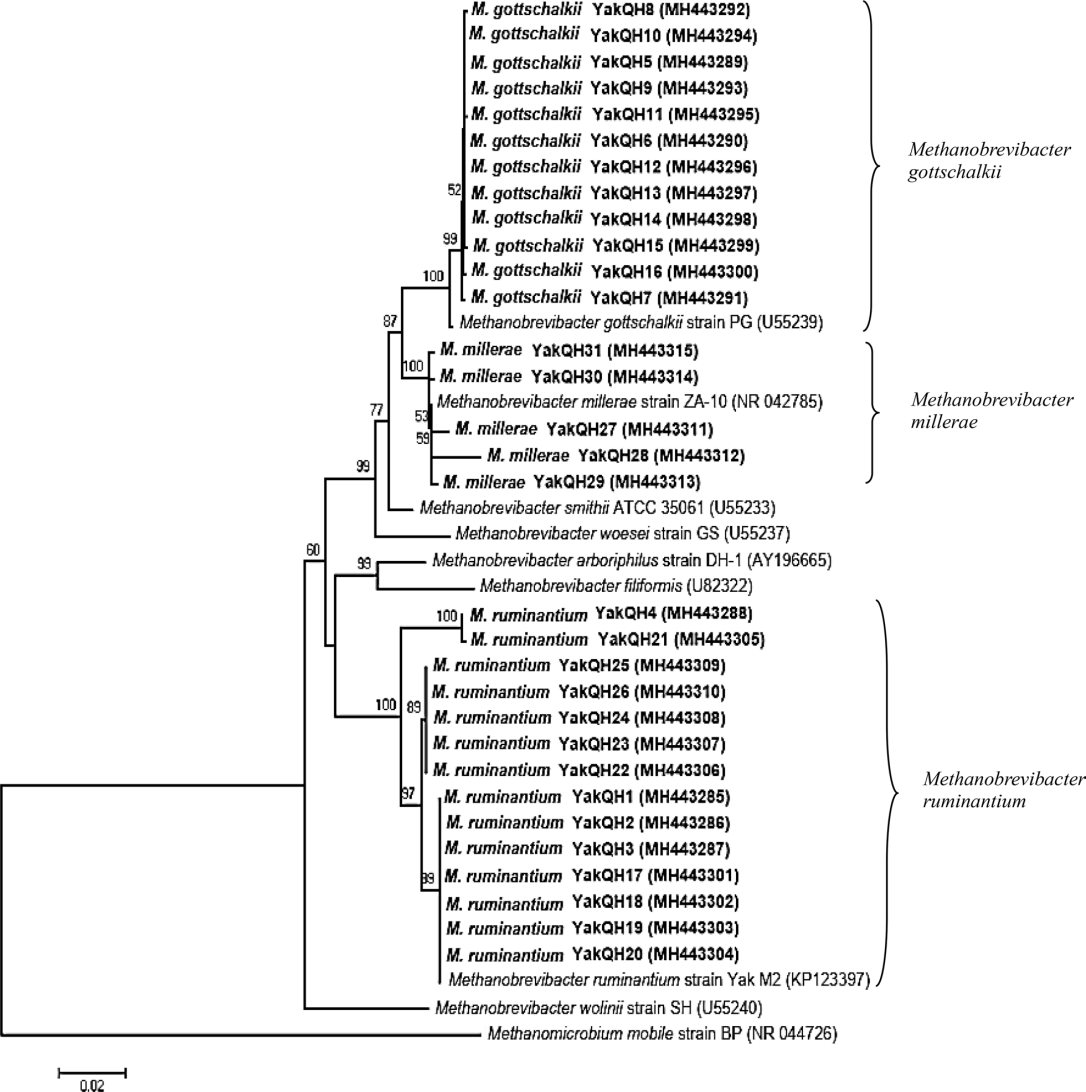
Phylogenetic tree of the 16S rRNA gene sequences of the methanogens isolates YakQH1–YakQH31 in the fungus–methanogen co-cultures. The topology of the tree was estimated by bootstraps based on 1000 replications. Bootstrap values more than 70% were shown on the major branch points. The scale bar corresponds to five changes per 100 positions. The 16S rRNA sequences determined in the present study and the closest relatives were marked in bold type. *Methanomicrobium mobile* BP (NR_044726) was used as an out group. GenBank accession numbers were given in brackets

A phylogenetic tree of the 16S rRNA gene sequences of methanogen isolates YakQH1-YakQH31 was constructed. *Methanomicrobium mobile* BP was used as an outgroup. The results showed that the 31 strains of methanogens included 3 species: *M. ruminantium*, *M. gottschalkii,* and *M. millerae* (Fig. 4).

### Screening of co-cultures

Among the 31 fungus–methanogen co-cultures, the 12 co-cultures of *N. frontalis* + *M.* *gottschalkii* grew most stably and rapidly and degraded wheat straw in anaerobic tubes to produce the highest total gas yields of 181–230 mL/g DM during the 5-day incubation, obviously more than other co-cultures. Particularly, the *N. frontalis* + *M. gottschalkii* co-culture YakQH5 was the most remarkable and was thus selected to determine its ability to biodegrade five types of roughage as substrates. The preservation number of the *N. frontalis* + *M. gottschalkii* co-culture YakQH5 was No. 19299 in the China General Microbiological Culture Collection Center (CGMCC).

### Enzyme profiles

During the 5-day incubation, in anaerobic bottles, the *N. frontalis* + *M. gottschalkii* co-culture YakQH5 degraded wheat straw, corn stalk, rice straw, oat straw and sorghum straw and showed the highest activity values of 12,910 mU/mL xylanase on wheat straw, 929 mU/mL CMCase and 1187 mU/mL FPase on rice straw, 10.5 mU/mL FAE and 245 mU/mL AE on corn stalk, and 20.5 mU/mL CAE on sorghum straw (Table 2).

**Table 2:** Fibrolytic enzyme of the *N. frontalis* + *M. gottschalkii* co-culture YakQH5 grown on the 5 sorts of substrates

Item	Wheat straw	Corn stalk	Rice straw	Sorghum straw	Oat straw
***Xylanase*** (mU/mL)					
Day2	4811 ^a^	3572 ^c^	2900 ^d^	4520 ^b^	3998 ^c^
Day3	10,703 ^a^	7812 ^c^	5926 ^d^	8995 ^b^	5911 ^d^
Day4	12,910 ^a^	9501 ^bc^	7673 ^c^	11,046 ^b^	8815 ^c^
Day5	8361 ^c^	10,598 ^a^	8235 ^c^	9985 ^ab^	9467 ^b^
Average	9196 ^a^	7871 ^c^	6183 ^c^	8637 ^b^	7048 ^c^
***CMCase*** (mU/mL)					
Day2	190 ^c^	173 ^c^	381 ^a^	150 ^c^	270 ^b^
Day3	285 ^c^	301 ^c^	570 ^a^	220 ^c^	460 ^b^
Day4	378 ^c^	396 ^c^	892 ^a^	386 ^c^	592 ^b^
Day5	450 ^d^	501 ^c^	929 ^a^	439 ^d^	721 ^b^
Average	326 ^c^	343 ^c^	693 ^a^	299 ^c^	516 ^b^
***FPase*** (mU/mL)					
Day2	260 ^b^	189 ^c^	367 ^a^	210 ^b^	217 ^b^
Day3	331 ^d^	396 ^c^	651 ^a^	298 ^c^	486 ^b^
Day4	469 ^d^	493 ^c^	970 ^a^	425 ^d^	573 ^b^
Day5	531 ^c^	558 ^c^	1187 ^a^	479 ^d^	683 ^b^
Average	398 ^c^	263 ^d^	795 ^a^	353 cd	489 ^b^
***FAE*** (mU/mL)					
Day2	2.0 ^a^	2.5 ^a^	1.9 ^b^	0.9 ^c^	1.7 ^b^
Day3	5.5 ^b^	6.4 ^a^	4.9 ^c^	3.1 ^d^	3.8 cd
Day4	8.9 ^a^	8.1 ^b^	9.1 ^a^	4.6 ^d^	5.9 ^c^
Day5	9.0 ^c^	10.5 ^a^	11.3 ^b^	7.5 ^d^	7.7 ^d^
Average	6.5 ^a^	6.9 ^a^	6.8 ^a^	4.0 ^c^	4.8 ^b^
***AE*** (mU/mL)					
Day2	59 ^d^	85 ^b^	80 ^b^	67 ^c^	99 ^a^
Day3	98 ^d^	145 ^ab^	130 ^b^	106 ^c^	157 ^a^
Day4	157 ^c^	198 ^a^	169 ^b^	177 ^b^	193 ^a^
Day5	183 ^d^	245 ^a^	175 ^d^	199 ^c^	208 ^b^
Average	125 ^c^	168 ^a^	139 ^b^	111 ^d^	113 ^d^
***CAE*** (mU/mL)					
Day2	0.7 ^c^	1.5 ^b^	0.8 ^c^	3.9 ^a^	0.9 ^c^
Day3	1.2 ^d^	2.8 ^b^	2.0 ^c^	8.8 ^a^	2.3 ^b^
Day4	2.0 ^d^	3.5 ^b^	2.6 ^c^	13.6 ^a^	3.1 ^b^
Day5	2.3 ^d^	4.9 ^b^	2.8 ^d^	20.5 ^a^	3.6 ^c^
Average	1.6 ^d^	3.2 ^b^	2.1 ^c^	11.7 ^a^	2.5 ^c^

CMCase, carboxymethyl cellulase; FPase, filter paper ase; FAE, ferulic acid esterase; AE, acetyl esterase; CAE, *p*-coumaric acid esterase; Day2-Day5: along the 5-day incubation; ^a^, ^b^, ^c^, ^d^ indicate statistical difference (*p* < 0.05)

### Fibre digestibility

**Fig. 5:**
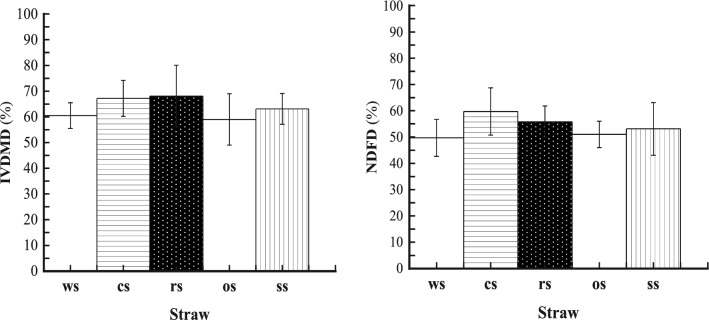
Fiber digestibility of the *N. frontalis* + *M. gottschalkii* co-culture YakQH5 grown on the 5 sorts of substrates. Left: In vitro dry matter digestibility (IVDMD); Right: Neural detergent fiber digestibility (NDFD). ws: wheat straw; cs: corn stalk; rs: rice straw; os: oat straw; ss: sorghum straw

During the 5-day incubation, in anaerobic bottles, the *N. frontalis* + *M. gottschalkii* co-culture YakQH5 degraded 60.5% of wheat straw, 67.2% of corn stalk, 68.1% of rice straw, 59.0% of oat straw, and 63.1% of sorghum straw (Fig. 5). Additionally, the co-culture YakQH5 had NDFD values of 49.5% on wheat straw, 59.7% on corn stalk, 55.8% on rice straw, 51.0% on oat straw, and 53.1% on sorghum straw (Fig. 5). The composition of the forages used as substrates before incubation was shown in Table 3.

**Table 3:** Composition of the forages used as substrates before incubation

	Corn stalk	Wheat straw	Rice straw Oat straw Sorghum straw	S.E.M
***Lignocellulose composition*** (g/kg DM)				
DM	940 ^b^	940 ^b^	953 ^a^ 931 ^b^ 950 ^a^	0.2
NDF	752 ^b^	783 ^a^	717 ^c^ 765 ^b^ 772 ^a^	3.2
ADF	426 ^b^	504 ^a^	445 ^b^ 410 ^c^ 421 ^c^	5.2
Cellulose	354 ^b^	419 ^a^	401 ^a^ 401 ^a^ 418 ^a^	5.0
Hemicellulose	322 ^a^	279 ^b^	273 ^b^ 278 ^b^ 269 ^b^	7.7
ADL	73 ^b^	87 ^a^	43 ^c^ 50 ^c^ 41 ^c^	1.4
***Phenolic acid*** (μg/g DM)				
PA	63 ^b^	96 ^a^	84 ^a^ 90 ^a^ 60 ^c^	3.6
VA	160 ^a^	156 ^a^	124 ^b^ 136 ^b^ 136 ^b^	4.0
FA	7530 ^a^	3757 ^c^	6058 ^b^ 2500 ^c^ 5625 ^b^	50.8
PCA	14,713 ^b^	5039 ^c^	7256 ^c^ 5105 ^c^ 26,507 ^a^	36.9

DM, dry matter; NDF, neutral detergent fiber; ADF, acid detergent fiber; ADL, acid detergent lignin; FA, ferulic acid; PCA, *p*-coumaric acid; VA, vanillic acid; PA, protocatechuic acid; S.E.M, standard error of mean

^a^, ^b^, ^c^, ^d^ indicate statistical difference (*p* < 0.05)

### Phenolic acid release

**Fig. 6:**
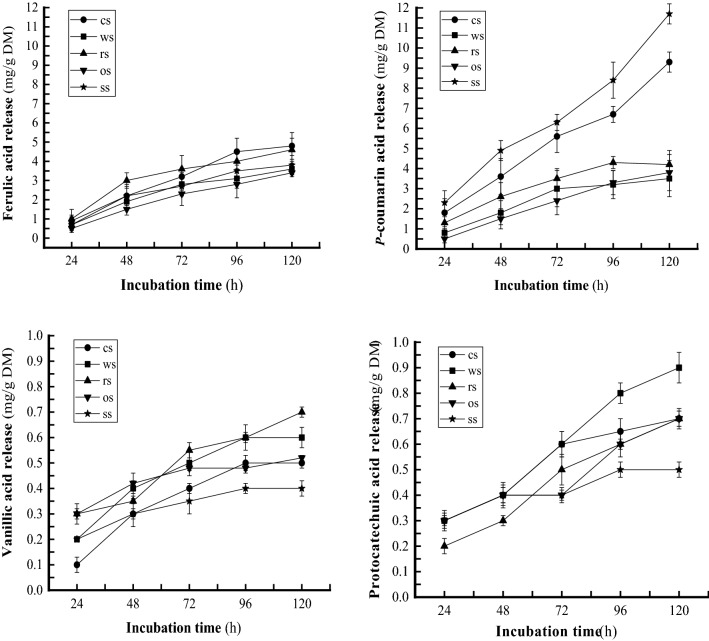
Releases of phenolic acids by the *N. frontalis* + *M. gottschalkii* co-culture YakQH5 grown on the 5 sorts of substrates. ● cs: corn stalk; ■ ws: wheat straw; ▲ rs: rice straw; ▼ os: oat straw; * ss: sorghum straw

During the 5-day incubation, in anaerobic bottles, the *N. frontalis* + *M. gottschalkii* co-culture YakQH5 degraded wheat straw, corn stalk, rice straw, oat straw, and sorghum straw to release FA 3.4–4.8 mg/g DM, PCA 3.4–11.7 mg/g DM, VA 0.4–0.7 mg/g DM and PA 0.5–0.9 mg/g DM, respectively. The peak values were as follows: FA 4.8 mg/g DM on corn stalk, PCA 11.7 mg/g DM on sorghum straw, VA 0.7 mg/g DM on rice straw, and PA 0.9 mg/g DM on wheat straw (Fig. 6).

### Fermentation end-products

**Fig. 7:**
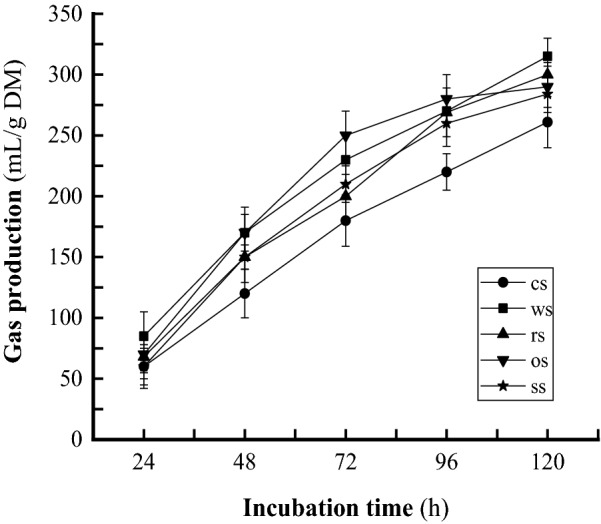
Gas production by the *N. frontalis* + *M. gottschalkii* co-culture YakQH5 grown on the 5 sorts of substrates. ● cs: corn stalk; ■ ws: wheat straw; ▲ rs: rice straw; ▼ os: oat straw; * ss: sorghum straw

During the 5-day incubation, in anaerobic bottles, the *N. frontalis* + *M. gottschalkii* co-culture YakQH5 degraded wheat straw, corn stalk, rice straw, oat straw and sorghum straw to produce high total gas yields (the gas was mainly composed of CH_4_) of 315 mL/g DM, 261 mL/g DM, 300 mL/g DM, 290 mL/g DM, and 284 mL/g DM, respectively (Fig. 7). Meanwhile, the *N. frontalis* + *M. gottschalkii* co-culture YakQH5 degraded these lignocellulosic materials to produce the highest end-product yields as follows: CH_4_ 4.6 mmol/g DM on wheat straw and acetate 8.6 mmol/g DM on rice straw (Table 4). The fungus *N. frontalis* YakQH5 degraded these lignocellulosic materials to produce maxima of H_2_ 3.9 mmol/g DM on wheat straw, formate 2.5 mmol/g DM on sorghum straw, acetate 5.5 mmol/g DM on rice straw, lactate 2.5 mmol/g DM on sorghum straw, and ethanol 45.8 mmol/g DM on wheat straw (Table 4).

**Table 4:** End-products produced by the *N. frontalis* + *M. gottschalkii* co-culture YakQH5 and the *N. frontalis* YakQH5 fungus grown on the 5 substrates

End-products	Wheat straw	Corn stalk	Rice straw	Oat straw	Sorghum straw
***N. frontalis* + *M. gottschalkii* Co-culture YakQH5**					
***CH***_***4***_ (mmol/g DM)					
Day2	1.5 ^a^	1.0 ^b^	1.7 ^a^	0.9 ^b^	1.2 ^b^
Day3	2.8 ^a^	2.6 ^a^	3.0 ^a^	2.1 ^b^	2.3 ^b^
Day4	3.8 ^a^	3.9 ^a^	3.5 ^a^	2.5 ^b^	2.9 ^b^
Day5	4.6 ^a^	4.2 ^a^	3.9 ^ab^	4.0 ^ab^	3.0 ^b^
Average	3.2 ^a^	2.9 ^a^	3.0 ^a^	2.4 ^b^	2.4 ^b^
***Acetate*** (mmol/g DM)					
Day2	2.0 ^b^	1.9 ^c^	2.1 ^b^	2.2 ^b^	2.9 ^a^
Day3	4.0 ^b^	4.0 ^ab^	4.6 ^a^	3.7 ^b^	5.0 ^a^
Day4	6.2 ^b^	6.0 ^b^	7.1 ^a^	5.9 ^c^	6.5 ^b^
Day5	8.0 ^b^	7.3 ^c^	8.6 ^a^	7.3 ^c^	8.2 ^b^
Average	5.1 ^b^	4.8 ^c^	5.6 ^a^	4.8 ^c^	5.7 ^a^
***N. frontalis* YakQH5 Fungus**					
***H***_***2***_ (mmol/g DM)					
Day2	0.5 ^a^	0.2 ^b^	0.4 ^a^	0.2 ^b^	0.5 ^a^
Day3	1.8 ^b^	1.0 ^d^	1.5 ^c^	2.0 ^a^	1.7 ^b^
Day4	2.8 ^a^	2.1 ^c^	1.9 ^d^	2.7 ^a^	2.5 ^b^
Day5	3.9 ^a^	2.5 ^c^	2.4 ^c^	3.0 ^b^	2.8 ^b^
Average	2.0	1.5	1.6	2.0	1.8
***Formate*** (mmol/g DM)					
Day2	0.4 ^b^	0.4 ^b^	0.6 ^a^	0.3 ^c^	0.6 ^a^
Day3	1.1 ^b^	1.1 ^b^	1.2 ^a^	1.1 ^b^	1.3 ^a^
Day4	1.7 ^b^	1.6 ^b^	1.6 ^b^	1.6 ^b^	1.9 ^a^
Day5	1.9 ^c^	2.0 ^b^	2.1^b^	1.9 ^c^	2.5 ^a^
Average	1.3 ^c^	1.3 ^c^	1.4 ^b^	1.2 ^d^	1.6 ^a^
***Acetate*** (mmol/g DM)					
Day2	1.5 ^a^	1.0 ^c^	1.5 ^a^	1.3 ^b^	1.2 ^b^
Day3	2.8 ^a^	1.9 ^c^	2.7 ^a^	2.0 ^b^	2.1 ^b^
Day4	4.0 ^a^	3.3 ^b^	4.1 ^a^	3.1 ^b^	3.2 ^b^
Day5	5.3 ^a^	4.1 ^c^	5.5 ^a^	4.4 ^b^	4.6 ^b^
Average	3.4 ^a^	2.6 ^b^	3.5 ^a^	2.7 ^b^	2.8 ^b^
***Lactate*** (mmol/g DM)					
Day2	0.6 ^c^	0.8 ^a^	0.5 ^d^	0.5 ^d^	0.7 ^b^
Day3	1.3 ^b^	1.5 ^a^	1.2 ^b^	1.2 ^b^	1.2 ^b^
Day4	1.9 ^b^	2.4 ^a^	1.8 ^b^	1.6 ^c^	1.7 ^c^
Day5	2.1 ^b^	2.4 ^a^	1.9 ^c^	1.9 ^c^	2.5 ^a^
Average	1.5 ^b^	1.8 ^a^	1.4 ^c^	1.3 ^c^	1.5 ^b^
***Ethanol*** (mmol/g DM)					
Day2	10.5 ^a^	8.0 ^b^	6.3 ^c^	9.5 ^b^	7.0 ^c^
Day3	30.3 ^a^	23.7 ^b^	19.1 ^c^	29.7 ^a^	25.0 ^b^
Day4 Day5 Average	37.1 ^a^ 45.8 ^a^ 30.9 ^a^	30.3 ^b^ 35.0 ^c^ 24.3 ^c^	24.7 ^c^ 27.9 ^d^ 19.5 ^d^	34.6 ^a^ 39.9 ^b^ 28.4 ^b^	30.1 ^b^ 33.4 ^c^ 23.9 ^c^

Day2-Day5: along the 5-day incubation; ^a^, ^b^, ^c^, ^d^ indicate statistical difference (*p* < 0.05)

## Discussion

### Diversity of the fungus–methanogen co-cultures from the rumen of grazing yaks

In this study, 31 natural fungus–methanogen co-cultures were first obtained from the rumen fluid of grazing yaks in spring in Qinghai Province, China, comprising 5 combination types: *N. frontalis* + *M. ruminantium*, *N. frontalis* + *M. gottschalkii*, *O. joyonii* + *M. ruminantium, C. communis* + *M. ruminantium*, and *C. communis* + *M. millerae*. In 2015, we isolated 20 natural fungus–methanogen co-cultures from the rumen fluid of grazing yaks in spring in a Wushaoling pasture of Tianzhu Tibetan Autonomous Prefecture in Gansu Province, China, including 4 combination types: *N. frontalis* + *M. ruminantium*, *O. joyonii* + *M. ruminantium*, *O. joyonii* + *M. millerae*, and *Piromyces* + *M. ruminantium* (Wei et al. 2015, 2016). Thus, there were many types of natural fungus–methanogen co-cultures in the rumen of grazing yaks. Furthermore, when compared to the reported natural fungus-methanogen co-cultures isolated from the rumen or faeces of ruminants and non-ruminants by Bauchop et al. (1981), Jin et al. (2011), Leis et al. (2014), Sun et al. (2014), Li et al. (2016) and Li et al. (2021) and grazing yaks by Wei et al. (2015, 2017), in this study, 3 new types of natural anaerobic fungus–methanogen co-culture combinations were first obtained from the rumen of yaks, namely: *N. frontalis* + *M. gottschalkii*, *C. communis* + *M. ruminantium*, and *C. communis* + *M. millerae.* These three types of fungus-methanogen co-cultures all included one fungus and one methanogen, and each methanogen coexisting with each fungus belonged to *Methanobrevibacter* sp., consistent with the natural fungus–methanogen co-cultures isolated from the rumen or faeces of ruminants and non-ruminants previously reported by Jin et al. (2011), Leis et al. (2014), Sun et al. (2014), Li et al. (2016), Li et al. (2021) and Wei et al. (2015, 2017).

Our study revealed that different combinations of natural fungus–methanogen co-cultures in the rumen of grazing yaks in different regions, probably because of the different types of wild herbages eaten by the grazing yaks in different areas, and these different types of natural fungus–methanogen co-cultures differed in their ability to degrade lignocelluloses. This suggests that there are new and abundant microbial resources for efficiently degrading lignocelluloses in the rumen of yaks grazing on the Qinghai-Tibet Plateau, which have not yet been fully explored.

### Roughage degradation by fungus–methanogen co-cultures from the rumen of grazing yaks

During the 5-day incubation, the *N. frontalis* + *M. gottschalkii* co-culture YakQH5 degraded the 5 kinds of roughages and showed degradation potential, including high lignocellulose-degrading enzyme activities, IVDMD 59.0%-68.1% (from oat straw to rice straw), NDFD 49.5%-59.7% (from wheat straw to corn stalk) and large amounts of FA and PCA releases, which are described in Sect. 3. Accordingly, we found that the degradation degrees of roughages were different. Ranked in terms of highest to lowest decomposition, the substrates were rice straw, corn stalk, sorghum straw, wheat straw, and oat straw, while in terms of highest to lowest degradation, they were corn stalk, rice straw, sorghum straw, oat straw, and wheat straw. The *N. frontalis* + *M. gottschalkii* co-culture YakQH5 degraded lignocelluloses by secreting main-chain degrading polysaccharide hydrolases (CMCase, FPase and xylanase) and side-chain degrading esterases (FAE, AE and CAE) with high activities, which could be key lignin-degrading enzymes in enhancing plant cell wall degradation. All these enzymes acted synergistically to effectively decompose lignocelluloses. The *N. frontalis* + *M. gottschalkii* YakQH5 degraded sorghum straw to release PCA 11.7 mg/g DM (70.1 μg/mL) as a result of the high PCA content in sorghum straw, consistent with the high activity of CAE when using sorghum stalk as a substrate, implying that the *N. frontalis* + *M. gottschalkii* YakQH5 from the rumen of Qinghai yaks can decompose lignin efficiently. Meanwhile, the *N. frontalis* + *M. gottschalkii* co-culture YakQH5 degraded wheat straw, corn stalk, rice straw, oat straw and sorghum straw to release very small amounts of VA and PA. The yields of VA and PA releases appeared unrelated to their contents in the roughages. Further study is needed to clarify this finding.

Among the 31 fungus–methanogen co-cultures, the *N. frontalis* + *M. gottschalkii* co-culture YakQH5 was screened out with only wheat straw as substrate by measuring gas production. In this case, the lignocellulose degradation and gas production are generally positive correlation.

The *N. frontalis* + *M. gottschalkii* co-culture YakQH5 degraded different roughages as substrates, the order of the lowest to highest IVDMD was: oat straw, wheat straw, sorghum straw, corn stalk, and rice straw; the order of the lowest to highest NDFD was: wheat straw, oat straw, sorghum straw, rice straw and corn stalk; and the order of the lowest to highest gas production was: corn stalk, sorghum straw, oat straw, rice straw and wheat straw. When the 5 kinds of roughages with different lignocellulose contents were used as substrates, there was not always a linear correlation between IVDMD, NDFD and gas production. This phenomenon may have been related to the different compositions of the five roughages. The lignocellulose degradation mechanism of the anaerobic fungi needs to be further studied to reveal the reason behind this phenomenon.

In 2015 and 2017, we first reported the natural fungus–methanogen co-culture *N. frontalis* + *M. ruminantium* Yaktz1 and *Piromyces* + *M. ruminantium* Yak-G18 that degraded straws with remarkable efficiency were isolated from the rumen of yaks grazing in Tianzhu Tibetan Autonomous County in Gansu Province of China (Wei et al. 2015, 2016, 2017). During the 7-day incubation, the *N. frontalis* + *M. ruminantium* co-culture Yaktz1 degraded 61.7% of wheat straw, 68.8% of corn stalk, and 71.9% of rice straw, with NDFD values of 56.0% on wheat straw, 61.7% on corn stalk, and 55.6% on rice straw, while exhibiting the highest enzyme activity values as follows: xylanase 12,500 mU/mL on wheat straw; FPase 430.3 mU/mL, FAE 11.4 mU/mL, AE 199.3 mU/mL and CAE 5.0 mU/mL on corn stalk, and FA release 24.1 μg/mL and PCA release 50.3 μg/mL on corn stalk as the peak values. Across the 7-day incubation, the *Piromyces* + *M. ruminantium* co-culture Yak-G18 degraded 60.5% of wheat straw, 65.0% of corn stalk, 65.9% of rice straw, 66.0% of Chinese wildrye, and 75.0% of alfalfa, with NDFD values of 40.8%–47.5% on the five substrates, showing peak values of xylanase activity ranging from 2750 to 5023 mU/mL (from alfalfa to Chinese wildrye), FPase ranging from 71.9 to 123.5 mU/mL(from rice straw to Chinese wildrye), and AE 66.3–118.1 mU/mL (from alfalfa to Chinese wildrye), releasing little FA and PCA. To date, 3 types of extremely effective fungus–methanogen co-cultures for straw degradation have been obtained from the rumen of yaks: the *N. frontalis* + *M. ruminantium* co-culture Yaktz1, the *Piromyces* + *M. ruminantium* co-culture Yak-G18, and the *N. frontalis* + *M. gottschalkii* co-culture YakQH5. According to degradation capability, the *N. frontalis* + *M. ruminantium* co-culture Yaktz1 and *N. frontalis* + *M. gottschalkii* co-culture YakQH5 showed the most prominent ability to degrade straws. The *N. frontalis* + *M. gottschalkii* co-culture YakQH5 from Qinghai yaks decomposed wheat straw, corn stalk, rice straw, oat straw, and sorghum straw to produce higher xylanase, FPase, and CAE activities than the *N. frontalis* + *M. ruminantium* co-culture Yaktz1 from Tianzhu yaks, other natural fungus-methanogen co-cultures (from rumen or faeces of ruminants and non-ruminants), and artificially mixed anaerobic fungus–methanogen co-cultures previously reported, with all kinds of roughages or fiter paper as substrates (Jin et al. 2011; Teunissen et al. 1992a, b; Wei et al. 2015, 2016, 2017). Specifically, the *N. frontalis* + *M. gottschalkii* co-culture YakQH5 showed FPase activity on corn stalk that was approximately 2.7 times higher than that of the *N. frontalis* + *M. ruminantium* co-culture Yaktz1 on corn stalk. The xylanase produced by *N. frontalis* + *M. gottschalkii* YakQH5 has good prospects for industrial application. Concurrently, the *N. frontalis* + *M. gottschalkii* co-culture YakQH5 could effectively degrade wheat straw, corn stalk and rice straw with IVDMD and NDFD values analogous to those of the *N. frontalis* + *M. ruminantium* co-culture Yaktz1 (Wei et al. 2016) but obviously higher IVDMD values than for other fungus–methanogen co-cultures from the rumen or faeces of ruminants and non-ruminants previously reported, with roughages as substrates (Jin et al. 2011). Values of 33.6%–53.1% IVDMD for wheat straw, corn stalk, bagasse, distiller’s dried grains with solubles (DDGS), wheat bran and rice straw by the *Piromyces* + *M. thaueri* CW co-culture from the rumen of goats; 26.8%–57.0% IVDMD for wheat straw, corn stalk, bagasse, DDGS, wheat bran, and rice straw by the *Piromyces* + *Methanobrevibacter* sp. Z8 co-culture from the rumen of goats; and 33.5%–48.3% IVDMD for rice straw by the *Anaeromyces* + *M. gottschalkii* strain PG co-culture from faeces of mules, the *Piromyces* + *M. gottschalkii* strain PG co-culture from faeces of mules, the *Piromyces* + *Methanobrevibacter* sp. Z8 co-culture from faeces of camel, the *Neocallimastix* + *Methanobrevibacter* sp. Z8 co-culture from feces of camel, and the *Piromyces* + *Methanobrevibacter* sp. 1Y co-culture from feces of buffalo, all after a 5-day incubation. The *N. frontalis* + *M. ruminantium* co-culture Yaktz1 degraded wheat straw, corn stalk, and rice straw to release the maximum values of FA 24.1 μg/mL and PCA 50.3 μg/mL on corn stalk, slightly lower than those of the *N. frontalis* + *M. gottschalkii* co-culture YakQH5 with corn stalk as substrate (Wei et al. 2016).

Our study showed that the *N. frontalis* + *M. gottschalkii* co-culture YakQH5 and the *N. frontalis* + *M. ruminantium* co-culture Yaktz1 from the rumen of grazing yaks degraded roughages more effectively than previously reported fungus-methanogen co-cultures from the digestive tract of herbivores, including ruminants and non-ruminants, and even some current industrial strains. This study also highlighted that a new-type fungus–methanogen combination, the *N. frontalis* + *M. gottschalkii* YakQH5, has been obtained from the rumen of Qinghai yaks, and it can superiorly degrade lignocellulosic materials. The *N. frontalis* + *M. ruminantium* co-culture Yaktz1 was isolated from yaks grazing in a Wushaoling pasture located in Tianzhu Tibetan Autonomous Prefecture in Gansu Province of China, an alpine meadow pasture with *Kobresia myosuroides* (*Villars*) *Foiri* as the main species, while the *N. frontalis* + *M. gottschalkii* co-culture YakQH5 was isolated from yaks grazing in Xinghai County located in Hainan Tibetan Autonomous Prefecture in Qinghai Province of China, where the pasture was alpine meadow with *Festuca Ovina L.* as the main species. It can be concluded that the combinations of fungus–methanogen co-cultures from the grazing yaks in different regions may vary, and these natural fungus–methanogen co-cultures had different characteristics and abilities to degrade lignocelluloses. Further studies on the host specificity or substrate specificity of anaerobic fungi are needed.

Meanwhile, the *N. frontalis* + *M. gottschalkii* co-culture YakQH5 degraded wheat straw straw, corn stalk, rice straw, oat straw, and sorghum straw to produce the highest yields of CH_4_ 4.6 mmol/g DM on wheat straw and acetate 8.6 mmol/g DM (55.7 mM) on rice straw. These are slightly higher yields than the CH_4_ and acetate yields produced by the *N. frontalis* + *M. ruminantium* co-culture Yaktz1 with wheat straw, corn stalk, and rice straw as substrates during the 7-day incubation; markedly higher than those produced by the *Piromyces* + *M. ruminantium* co-culture Yak-G18 on wheat straw, corn stalk, rice straw, Chinese wildrye, and alfalfa during the 7-day incubation; and higher than those produced by most of natural fungus–methanogen co-cultures from the rumen or faeces of ruminants and non-ruminants, and artificially mixed anaerobic fungus–methanogen co-cultures, with roughages, fiter paper, cellulose or glucose as substrates (Jin et al. 2011; Teunissen et al. 1992a, b; Li et al. 2016; Nakashimada et al. 2000; Wei et al. 2015, 2016, 2017).

After methanogen inhibition, the pure fungus *N. frontalis* YakQH5 degraded wheat straw, corn stalk, rice straw, oat straw and sorghum straw to produce the highest yields of H_2_ 3.9 mmol/g DM and ethanol 45.8 mmol/g DM (260.1 mM) on wheat straw, formate 2.5 mmol/g DM (15.0 mM) on sorghum straw, and lactate 2.5 mmol/g DM (15.0 mM) on sorghum straw. The yields of these end-products were generally higher than those produced by anaerobic fungi from the digestive tract of common ruminants and non-ruminants. The most interesting finding was that its ethanol yield was more greater than that produced by all reported anaerobic fungi with many kinds of roughages as substrates, even exceeding those of some industrial strains that produced ethanol (Jin et al. 2011; Sirohi et al 2013; Nagpal et al. 2011; Paul et al. 2010; Teunissen et al. 1992a, b; Sijtsma and Tan 1993; Thareja et al. 2006; Wei et al. 2016, 2017; Saye et al. 2021). Thus, the fungus *N. frontalis* YakQH5 is promising for use in the development of ethanol production.

### Prospects for the fungus–methanogen co-cultures from the rumen of grazing yaks

Anaerobic fungi have been studied for more than 40 years and are considered to play a crucial role in degrading lignocelluloses. Some methanogens can further enhance the anaerobic fungi's ability to degrade lignocelluloses. Anaerobic fungus–methanogen co-cultures degrade lignocellulosic materials to produce CH_4_, acetate and lignocellulose degradation enzymes with high activities, even exceeding those of some industrial strains (Teunissen et al. 1993; Cheng et al. 2018; Mountfort and Asher 1989; Solomon et al. 2016; Yang and Xie 2010; Chang and Park 2020). However, until now, anaerobic fungi have not been used for widespread industrial applications due to their strict anaerobic growth requirements, limited preservation methods, difficulty in scale-up, and genetic intractability. Recently, with the improvement of culture media, more species of anaerobic fungi have been discovered and a large number of their nucleotides and proteins have been sequenced (Chang et al. 2020; Hess et al. 2020).

In the present study, the 31 fungus–methanogen co-cultures were first obtained from the rumen of yaks grazing in Qinghai Province of China. These co-cultures included 5 combination types. Among them, during the 5-day incubation, the new-type combination *N. frontalis* + *M. gottschalkii* co-culture YakQH5 degraded 59.0%–68.1% of the DM and 49.5%–59.7% of the NDF of wheat straw, corn stalk, rice straw, oat straw and sorghum straw to produce CH_4_ (3.0–4.6 mmol/g DM) and acetate (7.3–8.6 mmol/g DM) as end-products and released the most FA (4.8 mg/g DM) on corn stalk, PCA (11.7 mg/g DM) on sorghum straw. The peak values of enzyme activitie were as follows: xylanase 12,910 mU/mL on wheat straw, FAE 10.5 mU/mL on corn stalk and CAE 20.5 mU/mL on sorghum straw. The *N. frontalis* + *M. gottschalkii* co-culture YakQH5 degraded roughages to produce higher xylanase, CMCase, FAE, AE activities, IVDMD, NDFD, and more CH_4_ and acetate yields without any pretreatment, than reported for natural fungus–methanogen co-cultures isolated from the digestive tract of ruminants and non-ruminants. This study convincingly proved our original hypothesis that Yak-derived ruminal fungus–methanogen co-cultures have evolved to possess high efficiency to degrade plant lignocellulose.

In follow-up studies, it will be useful strategy to further construct a large-scale continuous culture facility to produce natural complex lignocellulose-degrading enzymes, CH_4_ and acetate by the *N. frontalis* + *M. gottschalkii* co-culture YakQH5, or express its xylanase, CMCase, FEA, AE and CAE genes under aerobic conditions using molecular biology techniques to realize large-scale industrial production and application. The study on combined multiple omics analysis including genomics, transcriptomics, proteomics and metabolomics of the *N. frontalis* + *M. gottschalkii* co-culture YakQH5 will help to reveal its lignocellulose degradation mechanism. The fermentation process of the fungus–methanogen co-cultures is mainly carried out in the cytoplasm and hydrogenosomes, and the detailed fermentation mechanism also needs to be further studied and optimized. The fungus–methanogen co-cultures can efficiently degrade and change lignocelluloses into CH_4_ and acetate, representing potential for biological energy production that cannot be ignored. Therefore, screening the dominant combination of fungus–methanogen co-cultures from grazing yaks for the production of high-quality cellulase, hemicellulase, esterase and CH_4_ will have broad application prospects. Improving the preservation methods of anaerobic fungi with high activity will further promote their practical application in industrial and agricultural production. In the future, the study of the fungus–methanogen co-cultures from the rumen of Qinghai yaks will potentially be highly important to address feed shortages, agricultural wastes utilization, environmental pollution and energy crises.

## Data Availability

All data generated or analyzed during this study are included in this published article and the additional file.

## Abbreviations

ADF: Acid detergent fiber
ADFD: Acid detergent fiber digestibility
AE: Acetyl esterase
ATP: Adenosine triphosphate
ANOVA: Analysis of variance
BGI: Beijing genomics institute
BLAST: Basic local alignment search tool
CS: Corn stalk
CAE: Coumaric acid esterase
CMCase: Carboxymethyl cellulase
CGMCC: China General Microbiological Culture Collection Center
DGGE: Denaturing gradient gel electrophoresis
DDGS: Distillers dried grains with solubles
DM: Dry matter
FA: Ferulic acid
FAE: Ferulic acid esterase
FPase: Filter paper ase
GC: Gas chromatography
HPLC: High-performance liquid chromatography
ITS: Internal transcribed spacer
IVDMD: In vitro dry matter disappearance
NDF: Neutral detergent fiber
NDFD: Neutral detergent fiber digestibility
NCBI: National center for biotechnology information
OS: Oat straw
PBS: Phosphate-buffered saline
PCA: *p*-Coumaric acid
PCR: Polymerase chain reaction
PA: Protocatechuic acid
RS: Rice straw
SD: Standard deviation
SS: Sorghum straw
S.E.M: Standard error of mean
SEM: Scanning electron microscopy
VA: Vanillic acid
WS: Wheat straw
